# Towards Precise Positioning and Movement of UAVs for Near-Wall Tasks in GNSS-Denied Environments

**DOI:** 10.3390/s21062194

**Published:** 2021-03-21

**Authors:** Félix Orjales, Javier Losada-Pita, Alejandro Paz-Lopez, Álvaro Deibe

**Affiliations:** 1 Integrated Group for Engineering Research, Universidade da Coruña, 15403 A Coruña, Spain; adeibe@udc.es; 2 CITIC Research Center, Universidade da Coruña, 15071 A Coruña, Spain; javier.losada@udc.es (J.L.-P.); alpaz@udc.es (A.P.-L.)

**Keywords:** UAV, unmanned aerial vehicles, NWPS, indoor positioning systems, GPS denied, GNSS denied, autonomous vehicles

## Abstract

UAVs often perform tasks that require flying close to walls or structures and in environments where a satellite-based location is not possible. Flying close to solid bodies implies a higher risk of collisions, thus requiring an increase in the precision of the measurement and control of the UAV’s position. The aerodynamic distortions generated by nearby walls or other objects are also relevant, making the control more complex and further placing demands on the positioning system. Performing wall-related tasks implies flying very close to the wall and, in some cases, even touching it. This work presents a Near-Wall Positioning System (NWPS) based on the combination of an Ultra-wideband (UWB) solution and LIDAR-based range finders. This NWPS has been developed and tested to allow precise positioning and orientation of a multirotor UAV relative to a wall when performing tasks near it. Specific position and orientation control hardware based on horizontal thrusters has also been designed, allowing the UAV to move smoothly and safely near walls.

## 1. Introduction

Most UAVs use a Global Navigation Satellite System (GNSS) to determine their position. GNSS receivers are small, readily available, and easy to use. Working in areas where a GNSS constellation is not accessible represents a drawback for UAV use. For example, using UAV in civil and structural inspection applications is often limited to visual inspections, and GNSS is required [1,2]. The literature on this subject shows efforts made to develop aerial platforms capable of performing sound-based [2], contact-based [3], or hammering inspection [4], even though positioning and navigation for these platforms have not yet been fully developed in these conditions.

Operations carried out touching the wall or at a short distance away, in the same order of magnitude as the vehicle size, are a challenge for a UAV’s positioning control system. Proximity to walls or other objects distorts the aerodynamic currents generated by the UAV and causes changes in the UAV behavior, which, together with the proximity of the wall, increases the risk of collisions. Therefore, it is necessary to increase the control effort of the UAV under these conditions, and this also requires a more precise positioning system relative to the wall.

UAV positioning systems in applications without GNSS must be precise, with a high refresh rate and low latency. These positioning systems tend to be expensive. Moreover, its operation usually requires the deployment of precisely placed equipment and could require further calibration. These tasks tend to be cumbersome and time-consuming.

UAVs could change how some near-walls tasks are performed. Applications such as painting a high wall, installing anchor points without scaffolding, performing weld inspection in hard to reach areas, or carrying out wall thickness measurements. These tasks are usually expensive and dangerous, making the potential use of UAVs very valuable. However, the previously mentioned limitations restrict the use of UAVs in this kind of applications.

### 1.1. Positioning without GNSS

Positioning is one of the first and more significant steps required to develop an automatic or autonomous UAV. A positioning system can typically make use of direct measurement sources (beacons, ad hoc signals, etc.), inertial/dead-reckoning measurements (such as Inertial Measurement Units (IMUs) or Magnetic, Angular Rate, and Gravity sensors (MARGs)) and environmental correlation measurements (LIDAR, Ultra-wideband (UWB), etc.). Outdoor applications mostly rely on satellite-based positioning systems [5]. When a GNSS constellation is not available, the UAV’s navigation system needs to incorporate other ways to locate the vehicle.

Measurements from inertial sensors are relative readings and usually exhibit an accumulative positioning error. Environmental correlation measurement systems, on the other hand, deliver absolute measures. In this field, several wireless technologies and methods exist that can be employed to position a UAV flying indoors. Regarding the method used to evaluate distances, there are time-based, power-based, and angle-based approaches [6]. Time-based methodologies are among the most used, and most of them are based on Time of Arrival (ToA). Some variants differ on the synchronization method between transmitter and receiver and exhibit improved techniques for better accuracy [7]. Power-based approaches tend to be very sensitive to multipath or rebound generated by walls or obstacles. Received Signal Strength Indicator (RSSI) is among the best known, but the obtained measurements tend to be inaccurate [8]. Finally, angle-based approaches are uncommon as they require the use of specialized hardware.

Flying indoors usually has to be carried out with limited GNSS access or no access at all. In these cases, other Indoor Positioning Systems (IPS) must be used. Some of the characteristics of IPSs are determined by their physical layer. A survey of the most relevant IPSs currently available appears in [9] and more recently [10]. They include a comparison and a classification that can ease the IPS selection for the intended task. An IPS can be based on visible or invisible light (i.e., infrared), sound and ultrasound, magnetic fields, and RF. Widely used IPS technologies, such as Bluetooth [11], Wi-Fi, and UWB [12], fall into the last category.

### 1.2. Accurate Positioning and Movement Near Walls

To be viable in real-world scenarios, three key aspects need to be considered for the selection or development of a Near Wall Positioning System (NWPS) with UAV: low deployment complexity, high accuracy to avoid the obstacles in an enclosed and limited environment, and high refresh rate and low latency to control a fast and agile vehicle in a dynamically changing environment.

Navigation is another significant capability of an automatic or autonomous system, and it is deeply connected to the working environment. The requirements are different when navigating in a large, unobstructed environment or a confined space near walls. In the last case, moving from one point to another in a controlled and safe way implies dealing with obstacles, workspace limits, and aerodynamic distortions generated by the walls, the ceiling, or the ground. The orientation of the tool used to perform the intended task is another relevant aspect to consider, as it is linked with the UAV movement.

Multirotors are the preferred type of UAV for indoor applications because they can hold a static position and move indiscriminately in any horizontal direction. Horizontal movement is generated, in most cases, by tilting the vehicle. When a multirotor operates close to a wall, it is subject to changing aerodynamic forces. These forces vary with the distance and angle between the UAV and the wall. In general, these forces can change from repulsion to attraction with small changes in these parameters [13,14], making them unpredictable and, consequently, potentially leading to erratic behaviors. The ground and wall effect combination [15], as well as the tilt angle of the UAV [16], increase the effect of this phenomenon.

Both of the presented challenges, the positioning and control of the UAV, need to be addressed in order to obtain the full potential of the UAV in near-wall applications. Autonomous and automatic systems require reliable sensor inputs and adequate actuation outputs in order to be really effective. In addition, they need to be light-weight, simple, and low cost if they are intended to operate onboard little or medium-sized UAVs. These design limitations and requirements have been taken into account in the development of the proposed NWPS.

There are solutions, both commercial and in the research literature [17,18] that address these challenges. In most of these cases, they are concerned with detecting nearby walls or other objects and avoid colliding with them. The proposed solution, however, is different because it is focused on detecting the position and orientation of the wall relative to the UAV, navigating to reach closer to the wall, and making controlled contact with the tool to perform certain tasks.

Several tasks can be performed with a UAV that involve carrying some kind of probe to touch a wall in a controlled way. Tasks such as measuring the depth of carbonatation in concrete, sclerometry for determining the compressive strength of concrete, measuring dry film thickness, measuring metallic wall thickness, or detecting steel reinforcement bars in concrete structures, among others.

The intended task requires precise positioning of the tool or probe, making direct contact with the wall at a specific point and with a given angle. Both the position and orientation of the tool are essential factors for the success of the task. In the proposed architecture, the probe is rigidly attached to the body of the UAV. Therefore, the position and orientation of the UAV greatly influence the final result.

Regarding the positioning, what is presented in this document has focused on improving the accuracy in the distance and orientation estimations between vehicle and wall in the absence of GNSS signals. This requirement is critical to accomplish near-wall tasks. The new NWPS system merges two positioning solutions: LIDAR range finders and Ultra Wide Band (UWB) radio signals. At this point, this work has focused on indoor scenarios. However, this approximation could be applied outdoors after taking into account additional factors such as the weather conditions.

Regarding the control, multirotors need to tilt in pitch or roll axis to accomplish a horizontal displacement. This tilting action and the dynamic reactions related to it make difficult the precise positioning of the tool used to perform the task, it being a sensor or a manipulator. To overcome these problems, the proposed solution is based on the addition of small horizontal thrusters to the UAV and related hardware and software. With these thrusters, the UAV can make horizontal movements without tilting, thus easing the control of the tool.

The rest of the document has been structured as follows: Section 2 comprises the results of the literature review performed in relation to Indoor Positioning Systems and navigation near walls. In Section 3, the proposed positioning system based on UWB and LIDAR is presented. Section 4 explains in detail the proposed hardware system for horizontal movement close to walls. Section 5 summarizes the tests performed for both and highlights the more significant results. In the last section, a discussion of this work is presented.

## 2. Related Work

UAV research is a very active field with over 2500 scientific publications in the IEEE Xplore Digital Library during 2019 [19]. The two main challenges highlighted in the previous section are not an exception to this trend. This section will describe the most relevant efforts related to accurate interior positioning systems and navigation with multirotor UAVs near walls.

### 2.1. Precise Indoor Positioning

There are several alternatives available for indoor positioning. A survey and classification for all kinds of uses can be seen in [10]. In [20], there is a review of positioning systems for UAVs, as well as a discussion on their accuracy and characteristics. All in all, the prominent technologies used to perform accurate indoor position estimations (between 1 and 10 cm) are vision, ultrasonic, and UWB-based.

Vision-based systems as OptiTrack [21] or Vicon [22], based on motion capture, are very accurate, but their bulkiness and high cost make them inadequate. Simultaneous Location and Mapping (SLAM) is another option to consider. Thus, in [23] a UAV with stereo cameras is used, with a location error of less than 30 mm in the test set images, although in real-life scenarios higher errors are expected. In [24] an ultrasonic emitter is combined with Time of Flight (ToF) ranging cameras to improve the accuracy.

Regarding UltraWideBand (UWB) technology, in [25] a location system is used to perform indoor positioning with three drones at the same time, obtaining an error of around 250 mm. In subsequent work, this system is fused with SLAM techniques to improve the accuracy [26]. Similarly, works such as [27] follow the approach of merging UWB data with other sensors onboard the UAV platform, in this case inertial measuring units (IMU).

In the context of this work, UWB location systems present some advantages such as a high refresh rate and resistance to multipath propagation. Additionally, in comparison with methods such as SLAM, the UWB system requires less on-board data processing, saving weight and resulting in cheaper solutions.

### 2.2. UAV Movement Near Walls

Disturbances in aerodynamic forces generated by the UAV flight near walls need to be considered to perform the intended tasks successfully. The aerodynamic forces can be hard to predict. Small variations in UAV position or attitude can change interaction forces with the wall from repulsion to attraction. Both CFD techniques [28] and experimental data [29] have been analyzed to characterize these effects. They showed that flying very close to the wall can generate an attraction force to the wall and a rolling moment that tilts the UAV towards the wall. This problem can be mitigated using specific control algorithms on the UAV controller or making some hardware modifications. In [30], a controller that adapts in real-time against external disturbances is presented and tested in a near-wall flight.

Hardware modifications focus primarily on protecting the vehicle against damage caused by wall contacts, or in avoiding or controlling UAV tilting. Regarding hardware modifications, in [31] a shroud system used to absorb the impact energy is presented. In [32], a UAV with a spherical-shaped design comprised of an inner frame with a protective cage is described. For tilting control, there are systems capable of generating lateral forces without tilting the vehicle, such as in [33, 34] that present two platforms capable of changing the orientation of the thrusters while in flight. This solution offers more accurate handling of the UAV attitude and a faster response time. However, on the other hand, it needs a complex and heavier control system. In [35], a payload with two horizontal propellers is used to perform inspections in vertical walls.

The tilting of the UAV affects also the tool orientation, and most applications require a specific tool orientation. In [4], the tool is reoriented using articulations and actuators. However, these types of mechanical contraptions add weight and complexity and decrease flight time.

## 3. Proposed NWPS

This section presents the most relevant characteristics of the proposed NWPS. The system uses the combination of two positioning systems based on different technologies that give complementary information. The correct blend of both allows obtaining a better estimate of the UAV position in the planned work scenarios.

Near-wall tasks are a challenge for most IPS as they tend to offer the best accuracy around the center of the room and worsen as the vehicle approaches the walls. The proposed system combines a UWB based IPS for global positioning with a combination of onboard LIDAR range finders to improve near-wall accuracy. It is an evolution of the one presented in [36], with improved reliability and a generalized geometry for different LIDAR sensor configurations.

### 3.1. UWB-Based IPS

UWB positioning systems are among the most used for accurate indoor positioning. They are based on measuring the ToF of RF signals in the UWB frequency range (3.1 to 10.6 GHz). They have good refresh rate as well as better accuracy and robustness than other RF based solutions.

The working principle consists of various anchors located at fixed and known positions and a moving tag. The tag position is computed using the distances to the anchors. These distances are measured using a Two Way Ranging (TWR) technique. Then, a triangulation algorithm uses these distances to compute the absolute position of the tag. Using TWR instead of Time of Arrival (ToA), both outward and return ToF are measured. This strategy does not need a synchronization procedure between anchors and tags; however, on the other hand, it requires more time to perform the measurements and limits the maximum number of anchors and tags within the desired refresh rate.

Anchor count and their position are to be considered to obtain the best positioning accuracy. Three are the minimum required to compute a three-dimensional position estimation, but at least four are recommended, as the UAV or the people around can shade out some of them. However, too many anchors can lead to suboptimal results and delays in the triangulation process. The anchors should be positioned carefully to maintain the line of sight with the tag, for optimal performance.

The proposed NWPS uses a commercially available UWB positioning system known as Pozyx [37]. It is smaller than other solutions and has some technical advantages. It can use up to eight anchors simultaneously. According to manufacturer specifications, positioning error should be below 150 mm near the room center in most scenarios, with a refresh rate of 25 Hz. However, when flying near walls some artifacts and positioning errors are expected.

### 3.2. LIDAR-Based Positioning System

To improve positioning accuracy when flying near walls, a LIDAR based relative positioning system was developed. A set of m onboard LIDAR sensors measures punctual distances to the wall. A specifically developed algorithm uses these distances to estimate the UAV position and orientation relative to the wall. A detailed description of the algorithm follows.

The m LIDAR sensors are placed in the local (UAV) reference frame at the following coordinates:

(1) $$s_{i}=\left(x_{is},\;y_{is},z_{is}\right),i=1,\;2,\ldots ,m$$

The direction in which the sensor i is aiming is given by the unit vector as follows:

(2) $$a_{i}=\left(x_{ia},y_{ia},z_{ia}\right),\|a_{i}\|=1,i=1,\;2,\ldots ,m$$

Each sensor i. measures a distance $d_{i}>0$ to the wall. The points in the wall sensed by each of the sensors are at distances $d_{i}$ and directions $a_{i}$, from the point $s_{i}$. Thus, these points are at the following coordinates:

(3) $$p_{i}=\left(x_{ip},y_{ip},z_{ip}\right)=s_{i}+d_{i}\cdot a_{i}$$

Supposing the sensed surface (the wall) is a plane π, each of these m points should lie in it and, therefore, verify its equation in the form as follows:

(4) $$\frac{x_{ip}}{a}+\frac{y_{ip}}{b}+\frac{z_{ip}}{c}=1,i=1,\;2,\ldots ,m.\;$$

where a, b, and c are the intersection points of the plane π with the axes x,y, and z of the reference system, respectively. Naming r=1/a, s=1/b, t=1/c Equation (4) becomes

(5) $$x_{ip}\cdot r+y_{ip}\cdot s+z_{ip}\cdot t=1,i=1,2,\ldots ,m$$

There are m equations such as (5), one for each sensor. They form a system of linear equations (SEL) that can be expressed in matrix-vector form as follows:

(6) $$\left(\begin{array}{ccc} x_{1p} & y_{1p} & z_{1p}\\ x_{2p} & y_{2p} & z_{2p}\\ \vdots & \vdots & \vdots \\ x_{mp} & y_{mp} & z_{mp} \end{array}\right)\cdot \left(\begin{array}{c} r\\ s\\ t \end{array}\right)=\left(\begin{array}{c} 1\\ 1\\ \vdots \\ 1 \end{array}\right)$$

Naming the three terms of this equation as A, x**,** and b, respectively, Equation (6) becomes

(7) A⋅x=b 

Each equation in the SEL depends on a sensor measurement, $d_{i}$. These measurements are contaminated with noise; thus, in general, rank(A)=3, and rank(A|b)=4. Thus, the SEL will be overdetermined and inconsistent. Although there is no exact solution to this system, it is possible to compute a solution vector $x^{*}$. that is optimum in the sense of least squares minimization of the error e=‖A⋅x−b‖:

(8) $$\left\|A\cdot x^{*}-b\|=\underset{x}{\min}\|A\cdot x-b\right\|$$

When $A\in {\mathbb{R}}^{m\times 3}$. and rank(A)=3, the matrix $A^{T}\cdot A$. is regular, so pre-multiplying (7) by $A^{T}$ leads to the following:

(9) $$\left(A^{T}\cdot A\right)\cdot x^{*}=A^{T}\cdot b\;\to \;x^{*}={\left(A^{T}\cdot A\right)}^{-1}\cdot A^{T}\cdot b$$

Normalization of $x^{*}$ leads to the normalized equation of the optimum plane, as follows:

(10) $$R=\frac{r}{\left\|x^{*}\right\|},\;\;S=\frac{s}{\left\|x^{*}\right\|},\;\;T=\frac{t}{\left\|x^{*}\right\|},\;\;D=\frac{1}{\left\|x^{*}\right\|}\;\;\to \;\;R\cdot x+S\cdot y+T\cdot z=D.\;$$

where $n^{*}=\left(R,\;S,\;T\right)$ is a unit vector normal to the estimated plane $\pi^{*}$.

The computed plane $\pi^{*}$. is optimum. However, due to the noise that contaminates the distances $d_{i}$, the points $p_{i}$ do not lie in $\pi^{*}$. A global, normalized estimation of the error made by assuming the plan $\pi^{*}$. as the better solution can be made using the following:

(11) $$\sigma =\frac{\left\|A\cdot x^{*}-b\right\|}{\left\|x^{*}\right\|}$$

This error estimation can be used as a quality indicator for the goodness of the estimation of $\pi^{*}$ and to perform the fusion with the other information of the UWB.

**Figure 1:**
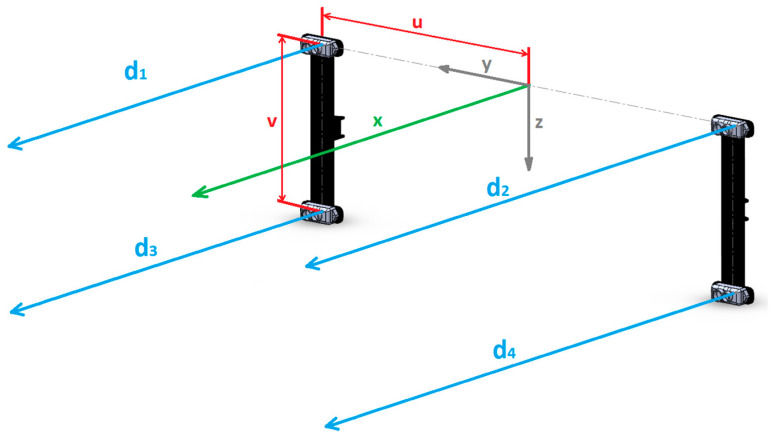
Frontal LIDAR-based range finders distribution in the UAV local reference frame.

In the current stage of development, the proposed LIDAR-based positioning subsystem has four sensors situated in the corners of a rectangle and facing the tool side of the UAV, the front in this case. They aim in the direction of the *X*-axis, as shown in Figure 1, and interact with the wall. The main requirements used for this LIDAR count and configuration were three: to allow position and orientation estimation of the UAV relative to the wall; to allow some failure tolerance; and to provide the quality estimator, σ. The selected sensors are “Benewake TFMini” and have an accuracy of ±6 cm from 0.3 to 6 m, ±1% from 6 to 12 m, and a 100 Hz refresh rate.

From Figure 1, $s_{1}={\left(0,u,0\right)}^{T}$, $s_{2}={\left(0,-u,0\right)}^{T}$, $s_{3}={\left(0,u,-v\right)}^{T}$, $s_{4}={\left(0,-u,-v\right)}^{T}$, and $a_{i}={\left(1,0,0\right)}^{T}$, so

(12) $$A=\left(\begin{array}{ccc} d_{1} & u & 0\\ d_{2} & -u & 0\\ d_{3} & u & -v\\ d_{4} & -u & -v \end{array}\right)$$

Solving (9) for $x^{*}={\left(r^{*},s^{*},t^{*}\right)}^{T}$,

(13) $$\begin{array}{c} r^{*}=r_{5}\\ s^{*}=-\frac{r_{3}\cdot r_{5}}{4\cdot u}\\ t^{*}=\frac{1}{v}\cdot \left(\frac{r_{2}\cdot r_{5}}{2}-1\right) \end{array}$$

where

(14) $$\begin{array}{c} r_{1}=d_{1}+d_{2}\\ r_{2}=d_{3}+d_{4}\\ r_{3}=d_{1}-d_{2}+d_{3}-d_{4}\\ r_{4}=\sum d_{i}{}^{2},i=1,..,4\\ r_{5}=\frac{4\cdot r_{1}}{4\cdot r_{4}-r_{3}^{2}-2\cdot r_{2}^{2}} \end{array}$$

Other simple mathematical relations compute the wall position and orientation relative to the UAV. This solution uses only simple algebraic operations, as shown in Equations (13) and (14). Therefore, it is easy to implement in a microcontroller. It allows real-time execution with a short delay time and minimal weight penalty for the UAV and generates, as has been shown, a quality measure of the estimation itself.

### 3.3. Data Fusion

Both the UWB and the LIDAR-based positioning subsystems deliver position estimations. However, the information that each one provides is different but complementary. The position from the UWB solution is absolute but without orientation information. The LIDAR subsystem delivers both distance and orientation information, but relative to the wall. The distance and orientation to the wall are critical parameters for near-wall tasks.

The fusion of this complementary information could offer an improvement over both subsystems but has to be adequately fused to unleash its full potential. The first step is to reference both measurements in the same coordinate system. Then, the data fusion itself can take place.

Both subsystems have been selected and designed to provide complementary information. However, there is no easy way to predetermine which one will offer the best estimation in real-life scenarios. For instance, when approaching the wall, UWB estimation should worsen, and LIDAR estimations should improve. However, unexpected situations can arise, and the estimations need to be evaluated in real-time to choose the optimal combination.

A specifically designed data fusion function based on fuzzy logic performs this task. This function uses the quality of the estimations from the LIDAR subsystem, σ, as the input parameter. Section 3.2 shows a detailed description of this function.

**Figure 2:**
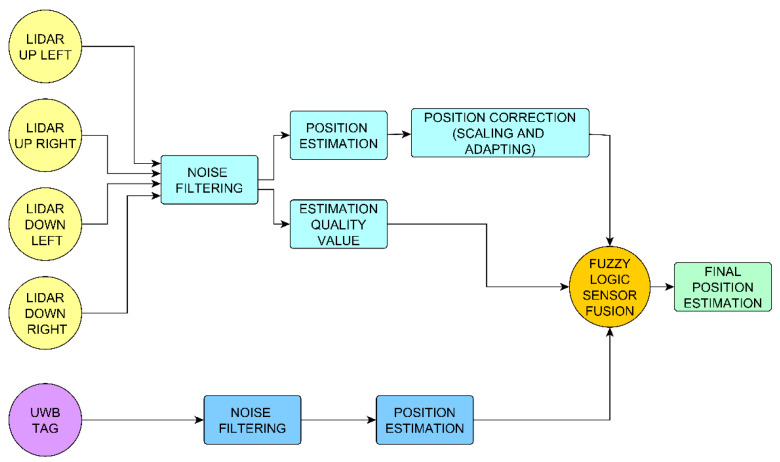
Conceptual diagram of the sensor fusion strategy based on fuzzy logic used for the position estimation.

Figure 2 shows a schematic representation of the proposed NWPS estimation process that is executed on a PIC32 microcontroller. The output is a position estimation with the potential to improve the measurements of both subsystems. It gives more weight to the LIDAR information, especially when the UAV is near a wall, and the LIDAR delivers better accuracy. The performance of this system has been evaluated with several tests, as described in Section 5.

## 4. Proposed Hardware for the Horizontal Movement of the UAV

This section presents the most relevant characteristics of the proposed modifications on the propulsion hardware of the multirotor.

One of the problems of near-wall movements is the aerodynamic distortions due to interactions between the wall and the prop wash. These distortions present a complex behavior, generating repulsive or attractive forces depending on the distance between wall and vehicle. Introducing the tilt angle of the UAV makes the interaction with the wall more unpredictable. This problem becomes worse when the distance to the wall becomes smaller. Additionally, flying near the floor or over other objects increases these forces due to the ground effect.

Another problem is related to the tool orientation. In several applications, the tool must interact with the wall at a certain angle. That is the case, for instance, of thickness measurement, which usually requires the tool to be positioned perpendicular to the measured surface. This task can be hard to accomplish with a conventional multirotor that needs to pitch or roll to change position.

**Figure 3:**
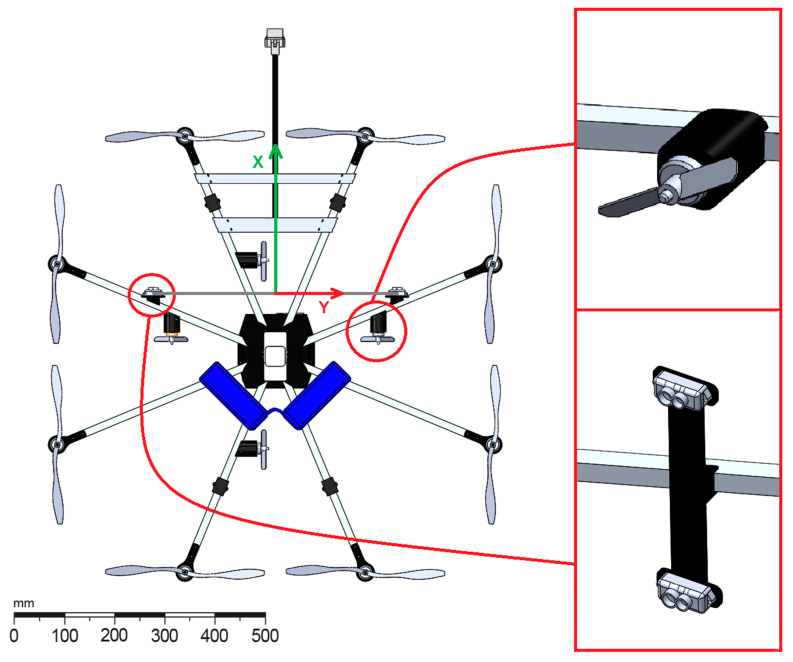
Placement of the LIDAR sensors and the horizontal movement hardware on the UAV platform.

To eliminate the tilt angle and allow a safer and more accurate horizontal movement, a horizontal hardware propulsion that consists of four small bidirectional motors and their propellers is added to the UAV. These thrusters are situated in a horizontal plane (XY), two of them facing the front, for forward/backward motion and the other two at a 90-degree angle for sideways motion, as shown in Figure 3.

In this case, a dummy tool was mounted at the front of the vehicle in order to make contact with the wall and simulate an inspection operation. It was mounted along the *X*-axis, between the front propellers, far enough to contact the wall while flying.

The four thrusters are controlled independently and share the power source of the UAV. A dedicated PIC32 microcontroller generates the control signals and is also responsible for all the NWPS calculations and fusion process explained in Section 3.3 The total weight added by the entire proposed system, including the propellers and associated electronics, is less than 200 g. With this configuration, the multirotor can move horizontally up to 3 m/s in X and Y directions without tilting. As vertical motion (in *Z*-axis) and yaw does not induce UAV tilting, with this strategy, the vehicle can yaw and displace in 3D without any induced tilting. The next section details the performance results of this propulsion system.

Lightweight and simplicity have a severe impact on the flying time of small UAVs. Keeping it light and simple helps in the final implementation in real-world scenarios. Both factors were considered from the initial design stages. The developed hardware is simple, so it does not require modifications to the original propulsion system. Only software configuration is needed to install it on a Commercial Off-The-Shelf (COTS) multirotor. Thus, the vehicle propulsion efficiency is not affected in the process. Furthermore, the little added weight enables its use even on small vehicles where other systems may not be feasible.

For all the tests in this paper, the proposed system was installed in an octocopter built using a HobbyKing X930 frame as a base. This frame has a glass fiber core structure, with aluminum arms for a total 895 mm of diameter. The main propulsion system is composed of eight Turnigy Aerodrive SK3 2836 brushless motors equipped with 305 × 105 mm propellers.

The power comes from two Lithium Polymer batteries with 3 cells in series and 5000 mAh each one, connected in parallel. All the embedded electronics are powered by the same battery using a dc-dc converter to reduce the voltage. A Raspberry Pi 3 microcomputer was also included to log and access easily to all the experimental data collected and synced by the PIC32 microcontroller using its integrated Wi-Fi connection.

### Control Architecture

A newly designed control architecture controls both the main propulsion system of the UAV and the proposed horizontal thrusters. The new control logic comprises different UAV behavior modes and allows the addition of new flight modes or modification of the existing ones to suit the task requirements.

A switch in the remote control lets the human pilot select the flight mode in real-time. In all flight modes, the UAV main rotors control altitude and yaw. In horizontal displacement flight modes, the vehicle moves without tilting, and the horizontal thrusters control the horizontal displacement of the UAV. In its current form, there are three flight modes:

- Manual. It allows the usual operation of the UAV, with all movements controlled by the main rotors. This mode is intended for fast positioning of the UAV and for taking off and landing maneuvers.
- Manual horizontal. It uses the thrusters for horizontal displacements, manually controlled by the user. The functions of the sticks in the remote control remain the same as in typical UAV operations. With this flight mode, a human pilot can be easily trained for precise horizontal motions, while the UAV main rotors are in charge of the rest of the movements.
- Auto horizontal. It is adjustable and customizable for the application. In its current form, one of the sticks of the remote control is used to control the distance to the wall and the sideways displacement speed of the UAV, while the control system uses the NWPS fusion algorithm to keep the vehicle perpendicular to the wall surface and at a distance set by the pilot.

## 5. Tests and Results

This section shows and analyzes the results of the tests designed and carried out to evaluate the performance of the NWPS and the hardware for navigation near walls.

All tests were conducted in the same laboratory, a free space with a vertical wall 4 m long, 4 m wide, and 6 m tall with a second-floor platform that eases the flight observation. The setup for the UWB system was the same for all the experiments and consisted of five anchors deployed around the workspace at different heights, following the manufacturers’ advice to obtain the best accuracy.

Three different test groups have been performed to evaluate all the proposed systems. The first one is comprised of the NWPS tests to evaluate the accuracy of the proposed positioning system. The second group assesses the horizontal movement hardware capabilities and advantages. Finally, a final test with both systems working together on a real UAV is also described and the results presented.

### 5.1. NWPS Tests Description

Two sets of tests were designed and performed to evaluate the accuracy of the proposed NWPS: one static and the other at a controlled speed.

On the static tests, the UAV was standing in five different positions over the intended working area, from 1 to 3 m from the wall in 0.5 m increments. Careful measurements resulted in precise values of distance to the wall in all static positions. The UWB and NWPS estimations were compared against that values, considered as ground truth.

The second set of tests consisted of wall approaching maneuvers. The dynamic tests have been designed considering the potential applications, and the approaching maneuver was selected as it is one of the most common for near-wall tasks. Wall approaching maneuvers were conducted at different, known and constant speeds. The UAV remained facing the wall in some of the tests. In the others, it maintained an angle of 20° to the wall. The comparisons made between the outputs from UWB and NWPS served to assess the improvements in the NWPS achieved with the incorporation of the LIDAR.

A conveyor belt with regulated speed controlled the approach to the wall in the dynamic maneuvers. A Hohner 58 series incremental rotary encoder with 2000 pulses per turn was installed in the 65 mm diameter drive pulley, leading to a final resolution for the estimated position of 0.1 mm. Two photocell sensors at known and fixed distances to the wall were also used to provide a precise ground truth reference for both the speed and position of the UAV.

The microcontroller onboard the UAV captured all the data from the sensors in real-time to keep a precise synchronization between the different streams and logged it into the Raspberry Pi 3 equipped onboard.

### 5.2. NWPS Tests Results

The results for both the static and dynamic tests described previously are presented and analyzed in order to offer a broad vision of the proposed NWPS performance in different conditions.

#### 5.2.1. NWPS Static Results

**Figure 4:**
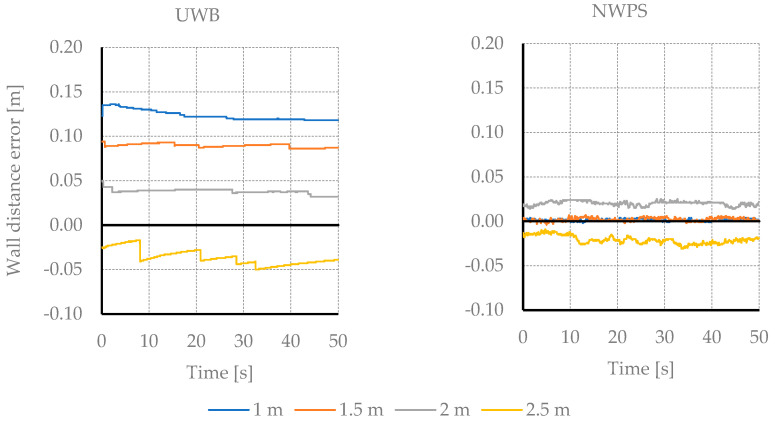
Results of the estimation of the distance to the wall in the static tests. Comparison between the Ultra-wideband (UWB) and the proposed Near-Wall Positioning System (NWPS).

Figure 4 shows the result of the wall distance estimation on the static tests. The initial distance is 1 m, and it is increased by 0.5 m in each measurement. Both measuring systems offer very little dispersion over the considered 5000 samples for each test. The average estimation error rises to 122 mm. As can be seen, the proposed NWPS provides improved accuracy and stability of measurements over the entire range of distances.

#### 5.2.2. NWPS Dynamic Results

**Figure 5:**
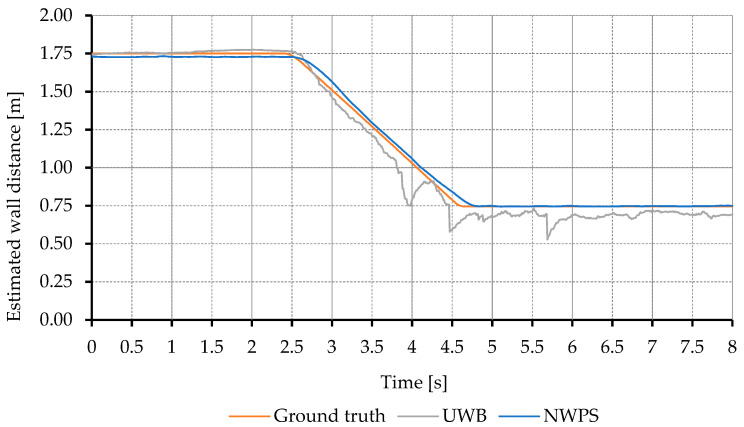
Estimation of the distance to the wall in perpendicular approaching maneuvers at 0.5 m/s. Comparison between conveyor belt measurements and the estimations from the UWB and the proposed NWPS.

A total of 20 tests were performed to evaluate the NWPS dynamic accuracy. A perpendicular and a 20-degree angle approach to the wall were tested. Each approach was repeated at five different speeds between 0.2 and 0.5 m/s using the conveyor belt and encoder system as a ground truth. All tests were repeated twice to reduce the chances of spurious errors. Figure 5 shows the distance estimation results for the fastest test performed perpendicular to the wall. As in the static tests, the UWB error increased with the proximity to the wall. As can be seen, the proposed NWPS behaves substantially better than the UWB alone.

**Figure 6:**
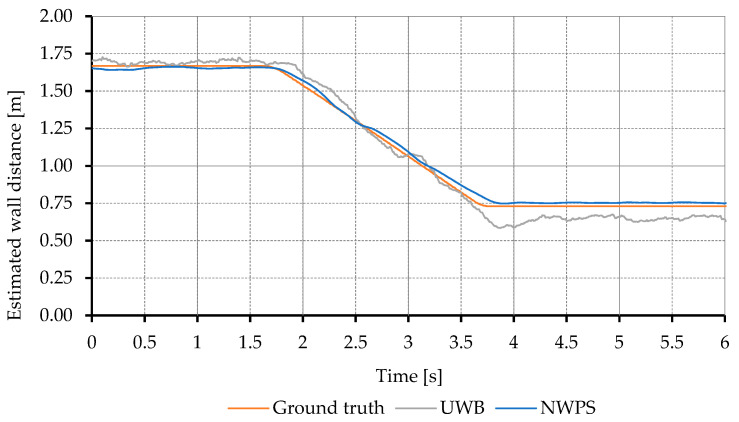
Estimation of the distance to the wall in 20-degree approaching maneuvers at 0.5 m/s. Comparison between conveyor belt measurements and the estimations from the UWB and the proposed NWPS.

Regarding the 20-degree angle tests, the results are very similar to the perpendicular approach with the UWB offering good estimations that worsen when the UAV is close to walls as can be seen in Figure 6. As in the previous case, the LIDAR information in the proposed NWPS improves the information from the UWB.

There are some sudden error spikes in the UWB estimation, which can also be seen in Figure 5. That kind of error can be potentially dangerous for fast control loops.

**Figure 7:**
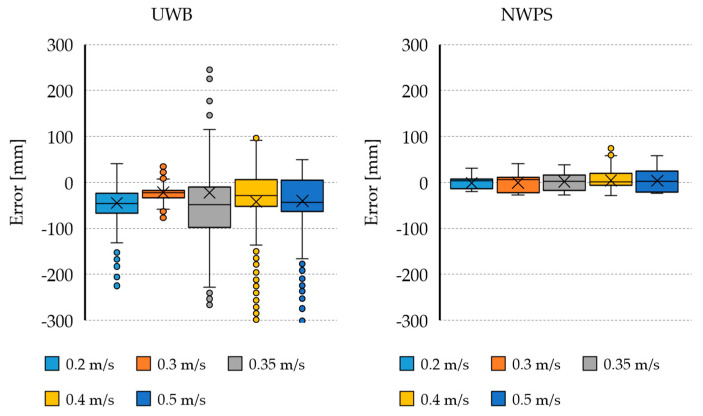
Estimation errors of the distance to the wall in the dynamic test, for the UWB and the proposed NWPS. Conveyor belt considered as ground truth.

Further analysis of the logged data can be seen in Figure 7, that shows a box plot of the estimation errors during dynamic tests compared to the conveyor belt ground truth, with data grouped by displacement speed in m/s. As expected, the error tends to increase with the speed, but the error magnitude and dispersion are higher for the UWB than for the proposed NWPS system. The high number of outliers in the UWB estimation evidence the spike-type errors seen in previous graphs.

### 5.3. Horizontal Movement Hardware Tests Description

A test was designed to demonstrate the effectiveness and evaluate the benefits of the horizontal position control hardware. This test, unlike all the others, was performed within an outdoor flight and was divided into two phases. In the first phase, the UAV performed a constant height linear movement in one direction and then returned to the origin. This phase was used to calculate the maximum horizontal speed that can be achieved using the horizontal thrusters. In the second phase, the UAV autonomously flew the same path as that of the previous phase and at the same speed. In this phase, however, only the main rotors were used.

This test was designed to be performed outdoors to be able to make high-speed movements without obstacles. Additionally, flying outdoors, GPS could be used as ground truth for the displacements and speeds. The test is used to reveal the expected enhancements of the proposed thruster-based navigation control schema. Moreover, the test shows the behavior of the proposed horizontal movement hardware in the face of disturbances such as wind or wind gusts.

### 5.4. Horizontal Movement Hardware Tests Results

The outdoor test flight was carried out in a UAV airfield, following all the legal and safety requirements. After the take-off, a flight was performed at a constant altitude of 20 m. The flight consisted of following a straight path back and forth. Using data from the onboard GPS, the estimated speed was 2.87 m/s one way and 2.8 m/s on the way back. The pitch angle during this displacement, measured using the UAV IMU was 1 degree.

The same speed was programmed for the second part of the test flight, but in this case, using the UAV main rotors. The UAV initially tilted 8 degrees in pitch to start the maneuver and then maintained the setpoint speed with a constant pitch angle of 6 degrees.

To perform wall-related tasks, the UAV could carry a tool, usually at the end of a pole. The tool movement created by this pitch angle is directly proportional to the distance of the tool to the multirotor center of rotation. This distance is 850 mm for the multirotor and dummy tool used in these tests. With that distance and the measured tilt angle, the resulting vertical tool displacement can be estimated at 118 mm for the start of the maneuver and 89 mm for the continuous movement. These unforeseen displacements make the precise positioning of the tool difficult.

This test demonstrates the capabilities and advantages of the proposed propulsion system to successfully move the multirotor at reasonably high speeds without undesired pitch or roll movements. It also tries to illustrate the tilt-induced tool movement, problematic when performing near-wall tasks, that the proposed navigation system can overcome.

### 5.5. Description of the Final Test

A final test was designed to verify the compliance with the expected capabilities of both the proposed NWPS and the navigation control system. The test consisted of real indoor flight in the absence of GNSS signals, with a UAV using the proposed NWPS and navigation control hardware. The test was run in four phases:

1. Takeoff and UAV stabilization at a distance of 2.5 m away from the wall.
2. Controlled approach maneuver to the wall using the horizontal thrusters, and the position estimation generated by the NWPS.
3. Stationary flight at a distance of 1 m to the wall (0.2 m from the tip of the tool).
4. Return to the origin and landing.

### 5.6. Final Test Results

**Figure 8:**
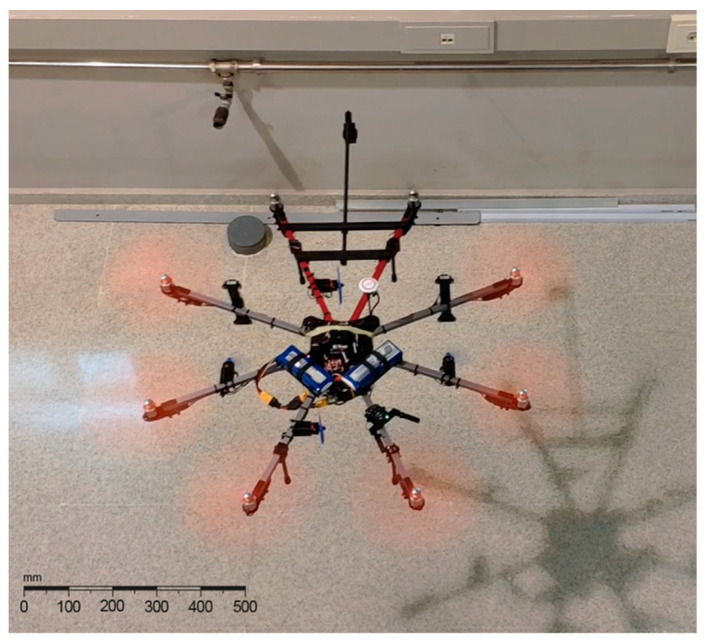
UAV flying close to a wall during the final test. The proposed NWPS and horizontal movement hardware are used to hover at a fixed distance to the wall.

**Figure 9:**
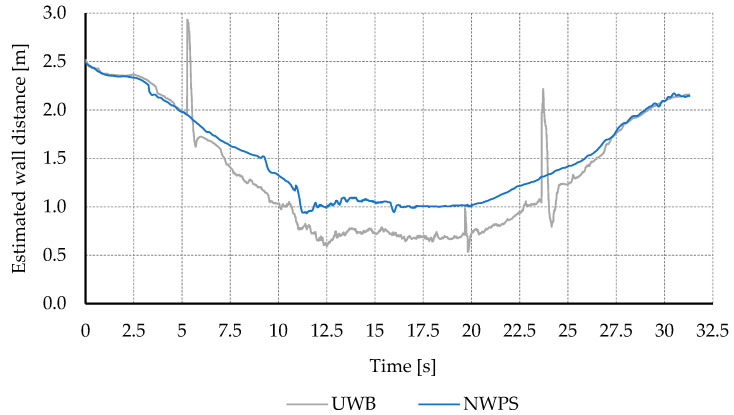
Estimation of the distance to the wall in an indoor flight test. Comparison between results from the UWB and the proposed NWPS.

The test was performed maintaining the same workspace and UWB setup and the maneuvers were recorded from an elevated platform to better estimate distances to the wall. A photogram of the recorded flight can be seen in Figure 8: 

Figure 9 shows the estimation of the distance to the wall, for both the proposed NWPS and the UWB. The same behavior is observed as in previous tests. The UWB offered good accuracy in general, with both systems estimations almost overlapping. However, the UWB system showed some spike-shaped errors and in the experiments performed underestimated the distance to the wall when flying close to it.

This underestimation problem could be caused by multipath propagation. When the vehicle is close to the wall part of the UWB signal travels directly, while some bounces off the wall, travelling a longer path. This may cause the UWB to estimate that the vehicle is further away from the anchors than what it really is and, consequently, closer to the wall.

## 6. Discussion

This paper presents a new near-wall positioning system (NWPS) that estimates the position of a multirotor UAV touching a wall or flying at a short distance away in the same order of magnitude as the vehicle size in GNSS-denied scenarios. A new strategy for controlling the position of the UAV and its associated tool near walls is also presented.

The proposed NWPS is based on the combination of UWB sensors and LIDAR range finders. It has been developed to improve the robustness and accuracy of commercially available IPS, particularly near walls. This NWPS adds very little weight to the UAV, and the selected estimation algorithm approach allows the computation of the position estimation using lightweight and low-cost microcontrollers. The range of the system and its accuracy is directly related to the LIDAR capabilities. The selected LIDAR sensors allow a range of up to 12 m. For the best accuracy, direct vision of the wall is required from all sensors.

The tilting of the UAV induces troublesome displacements of the working tool. This tilting can create turbulence and dangerous interaction forces with the wall. This paper presents a new strategy that allows the control of the UAV position without tilting. This strategy is based on the use of small thrusters and associated control algorithms and hardware.

Both systems have been tried separately in various tests to verify their correct operation and probe their advantages. Then, both systems were tried together, in a set of real flight tests on a GNSS denied indoor environment, flying near walls, with successful results. The outcome of the present work walks a few steps towards autonomous UAV usage in near-wall tasks. The use of UAV in these situations can reduce the operational costs of expensive processes, i.e., civil and structural inspections.

## Data Availability

Not applicable.
